# Sleeping position affects the hairline recession in male patients with androgenetic alopecia

**DOI:** 10.1016/j.jpra.2025.12.028

**Published:** 2026-01-03

**Authors:** Yi Wu, Tingru Dong, Tatiana Gomez Gomez, Yeqin Dai

**Affiliations:** a Zhejiang Chinese Medicine University affiliated Hangzhou Third People's Hospital, Hangzhou Third People’s Hospital, Hangzhou, Zhejiang, China; b Dr. Phillip Frost Department of Dermatology and Cutaneous Surgery, University of Miami Miller School of Medicine, Miami, Florida, USA; c T.H. Chan School of Public Health, Harvard University, ECPE-PPCR, Boston, MA, USA

**Keywords:** Androgenic alopecia, Hairline recession, Asymmetry, Sleeping position, Combing direction, Hair follicle

## Abstract

Clinical observations indicate that some male patients with androgenic alopecia show varying degrees of hairline recession in the frontal and temporal regions on both sides, often with noticeable asymmetry between the left and right, which suggests a potential link to certain lifestyle habits. In this study, 161 male patients with androgenic alopecia were included, all exhibiting an “M”-shaped hairline. Patients' sleeping positions and combing directions were recorded and the areas of hairline recession in the frontal and temporal regions were calculated. Analysis of variance showed a significant difference in the ratio of the left and right receding hairline areas among patients with different sleeping positions (left lateral 1.19 ± 0.15 vs. right lateral 0.90 ± 0.10 vs. supine 1.00 ± 0.15 vs. no fixed position 1.00 ± 0.14, *F* = 24.84, *p* < 0.01). However, no significant difference in hairline recession was observed between patients who combed their hair to the left and those who combed to the right (*p* = 0.47). In conclusion, sleeping position may be an independent factor that influences the pattern of hairline recession of male patients with androgenic alopecia, particularly those with “M”-shaped hairlines, whereas combing direction appears to have no impact.

Androgenic alopecia is a common form of non-scarring hair loss characterized by the miniaturization and progressive thinning of hair. In men, this condition typically presents as a receding hairline at the forehead, with reduced hair density in the frontal and temporal regions on both sides, often forming an “M”-shaped pattern.^1^ Various factors contribute to androgenic alopecia, including genetic, age, sleep, diet, sun exposure, and scalp health.^2^, ^3^, ^4^ Clinical observations indicate that some male patients show varying degrees of hairline recession in the frontal and temporal regions on both sides, often with noticeable asymmetry between the left and right. Azar et al. revealed that the bald frontotemporal area on the right was larger than its counterpart on the left in most male patients with androgenic alopecia.^5^ This suggests a potential link to certain lifestyle habits. The current study aims to analyze the correlation between sleeping position, combing direction, and the degree of hairline recession in the frontal and temporal regions of male patients.

The study included 161 male patients with androgenic alopecia, all exhibiting an “M”-shaped hairline and no history of hair loss treatment (systemic or local administration). All included patients had no history of obesity, sleep apnea, respiratory diseases or other conditions that may influence sleeping position. The mean age of all participants was (32.29 ± 7.35) years, with a mean hair loss duration of (4.49 ± 2.23) years and a mean daily sleep duration of (7.11 ± 0.74) hours per night. Patients were inquired about their sleeping positions (left lateral, right lateral, supine, or no fixed position) and combing directions (left, right, forward, or no combing) by one researcher. The area of hairline recession in the frontal and temporal regions on both sides was measured by another researcher. For consistency, the area of hairline recession was defined as the region enclosed by a line connecting the anterior point (T) of the temporal peak on both sides, the lowest point (C) of the frontal hairline, and the hairline of the frontal and temporal regions (Figure 1). The posterior displacement of the hairline in these regions was calculated using grid paper.

**Figure 1:**
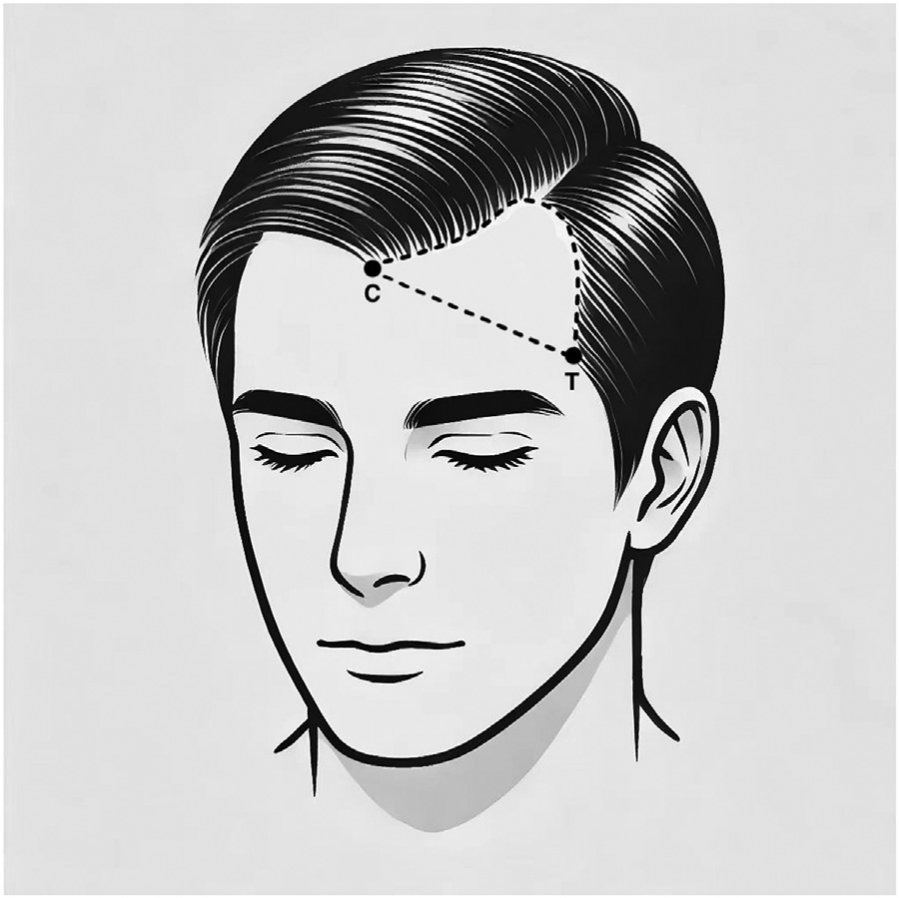
The area of hairline recession was defined as the region enclosed by a line connecting the anterior point (T) of the temporal peak on both sides, the lowest point (C) of the frontal hairline, and the hairline of the frontal and temporal regions.

Paired t-test analysis within groups showed that patients who habitually slept on their left side had a significantly larger area of hairline recession in the left frontal and temporal regions compared to the right (*p* < 0.01). Conversely, those who primarily slept on their right side exhibited more pronounced recession on the right side (*p* < 0.01). No significant difference was observed in hairline recession areas on both sides among patients who slept in the supine position (*p* = 0.78) or had no fixed sleeping position (*p* = 0.65). To further assess the impact of sleeping position on hairline recession, we introduced the ratio of the left and right receding hairline areas. Analysis of variance showed a significant difference in the ratio of the left and right receding hairline areas among patients with different sleeping positions (left lateral 1.19 ± 0.15 vs. right lateral 0.90 ± 0.10 vs. supine 1.00 ± 0.15 vs. no fixed position 1.00 ± 0.14, *F* = 24.84, *p* < 0.01). However, no significant difference in hairline recession was observed between patients who combed their hair to the left and those who combed to the right (*p* = 0.47). There was no significant interaction effect between sleeping position and combing direction. (*F* = 0.41, *p* = 0.93) (Table 1).

**Table 1:** The correlation between sleeping position, combing direction, and the degree of hairline recession in the frontal and temporal regions of male patients with androgenic alopecia.

Basic characteristics				
Age (years, mean ± SD)				32.29 ± 7.35
Duration of hair loss (years, mean ± SD)				4.49 ± 2.23
Nightly sleep duration (hours, mean ± SD)				7.11 ± 0.74

Sleeping position				

	Left lateral	Right lateral	Supine	No fixed position

N (%)	31 (19.25)	46 (28.57)	61 (37.89)	23 (14.29)
Paired t-test (t, *p*)	8.21, <0.001	−6.63, <0.001	−0.78, 0.78	−0.46, 0.65
Ratio (mean ± SD)	1.19 ± 0.15	0.90 ± 0.10	1.00 ± 0.15	1.00 ± 0.14
Two-way ANOVA	*F* = 24.84, *p* < 0.001 S			
Multiple comparisons	Left lateral vs. Right lateral, *p* < 0.001 S Left lateral vs. Supine, *p* < 0.001 S Left lateral vs. No fixed position, *p* < 0.001 S Right lateral vs. Supine, *p* < 0.001 S Right lateral vs. No fixed position, *p* = 0.003 S Supine vs. No fixed position, *p* = 0.94 NS			

Combing direction				

	Left	Right	Forward	No combing

N (%)	23 (14.29)	38 (23.60)	44 (27.33)	56 (34.78)
Paired t-test (t, *p*)	0.80, 0.43	−0.50, 0.62	−1.46, 0.15	0.42, 0.68
Ratio (mean ± SD)	1.03 ± 0.15	1.00 ± 0.16	0.98 ± 0.15	1.03 ± 0.19
Two-way ANOVA	*F* = 0.59, *p* = 0.62 NS			
Multiple comparisons	Left vs. Right, *p* = 0.47 NS Left vs. Forward, *p* = 0.15 NS Left vs. No combing, *p* = 0.92 NS Right vs. Forward, *p* = 0.41 NS Right vs. No combing, *p* = 0.43 NS Forward vs. No combing, *p* = 0.08 NS			

There was no significant interaction effect between sleeping position and combing direction (*F* = 0.41, *p* = 0.93). Ratio, left receding hairline areas/right receding hairline areas.

SD, standard deviation; S, significant; NS, non-significant.

Prior study revealed that hairline regression in patients with androgenic alopecia was asymmetrical, a disorder-associated phenomenon with unknown biological causality.^5^ This study demonstrates that sleeping position may be an independent factor that influences the pattern of hairline recession, whereas combing direction appears to have no impact. It is postulated that prolonged scalp compression during sleep may restrict blood circulation, reducing the blood supply to hair follicles and negatively affecting hair growth. Additionally, residual sebum from pillows may accumulate on the scalp, potentially obstructing hair follicle function. These findings underscore the effect of sleeping position on the progression of hairline recession in male patients with androgenetic alopecia, particularly those with “M”-shaped hairlines, and provide practical guidance for patients aiming to prevent further progression of unilateral hair loss. However, this conclusion of this study is mainly drawn from clinical observation, and further investigations are needed to elucidate the underlying mechanisms involved.

## Funding

This research is granted from Hangzhou Biomedical and Health Industry Development Support Project (No. 2021WJCY314).

## Ethical approval

Reviewed and approved by the Medical Ethics Committee of Hangzhou Third People's Hospital.

## Patient consent

The patients in this manuscript have given written informed consent to publication of their case details.

## Declaration of competing interest

None declared.
